# Assessment and Clinical Classification of Accommodative Dysfunctions in Non-presbyopic Patients: A Narrative Clinical Review

**DOI:** 10.7759/cureus.105684

**Published:** 2026-03-23

**Authors:** Andronikos Chrysanthopoulos, Evangelos Pateras, Athina Plakitsi

**Affiliations:** 1 Optics and Optometry, Biomedical Department, University of West Attica, Athens, GRC

**Keywords:** accommodation facility, accommodative dysfunction, assessing accommodative function, dynamic accommodation, lag/lead of accommodation, non-presbyopic patient, non-strabismic binocular vision anomalies

## Abstract

This review aims to provide a summary of the present knowledge in the assessment and classification of accommodative dysfunctions in non-presbyopic patients, with special emphasis placed on the impact of the assessment techniques and protocols employed in the measurement of the dysfunctions. The most commonly employed clinical and objective techniques in the assessment of accommodative dysfunctions are discussed, in addition to the major limitations and diagnostic biases associated with the techniques. The major accommodative dysfunctions include insufficiency, infacility, spasm, fatigue, and paresis, while the most striking limitations in the assessment of these dysfunctions include inconsistencies in the assessment techniques and protocols employed, as well as the diagnostic criteria and norms. A pragmatic approach for the diagnosis of the dysfunctions is proposed with the aim of enhancing the specificity of the diagnostic techniques employed in the assessment of the dysfunctions. Standardization of the techniques and protocols employed in the assessment of the dysfunctions, in addition to the integration of objective dynamic techniques in the assessment of the dysfunctions, may help in the elimination of the diagnostic biases associated with the assessment techniques employed. This narrative review was developed using recent peer-reviewed articles retrieved from major scientific databases, including PubMed, ScienceDirect, Wiley Online Library, and Scopus, with emphasis on clinically relevant and recent publications.

## Introduction and background

Vision is a complex function that is closely linked to broader physiological processes and is continuously influenced by environmental stimuli. A key mechanism underlying this interaction is the autonomic nervous system, which modulates several ocular functions through its parasympathetic and sympathetic divisions. Parasympathetic innervation primarily mediates pupillary constriction and accommodation via projections to the sphincter pupillae and ciliary muscle, whereas sympathetic innervation supplies the dilator pupillae and exerts a slower, more limited influence on accommodation. Together, these pathways contribute to the regulation of ocular responses under changing visual conditions [1].

Accommodation refers to the eye’s capacity to adjust the focusing power of the crystalline lens so that images of objects at different distances remain sharp on the retina [2-4]. This mechanism relies on the coordinated action of the ciliary muscle, zonular fibers, and the lens itself: when the ciliary muscle contracts, the zonules relax, the lens becomes more curved, and the eye’s optical power increases for near viewing. The accommodative response does not occur instantly, but follows a brief delay that is generally reported to be around 300-500 ms [3]. When there is no active visual demand, the accommodative system settles into a tonic resting position, which is shaped by parasympathetic input and can vary with factors such as age, refractive error, lighting conditions, and recent visual activity [5-7]. Prolonged near work may also influence tonic accommodation and lead to temporary changes in accommodative performance [6]. Accommodation plays a central role in maintaining clear and comfortable near vision, and dysfunctions within this system can produce symptoms such as asthenopia, intermittent blur, difficulty maintaining near tasks, and reduced reading performance. In non-presbyopic individuals, accommodative dysfunctions are therefore clinically important, yet their evaluation and classification remain challenging. One reason for this is the lack of diagnostic consistency, as test results are affected by the assessment method, target characteristics, viewing conditions, endpoint criteria, and the normative cutoffs used to define abnormal findings. In addition, recent advances in objective diagnostic technologies, including wavefront sensing and open-field autorefractors, have improved the precision of accommodative assessment and provided new opportunities for more detailed functional characterization of accommodative disorders.

This narrative review aims to summarize current evidence regarding the assessment and classification of accommodative dysfunctions, with a particular focus on the methodological factors that affect diagnostic accuracy. By examining commonly used clinical and objective methods and emphasizing the main sources of diagnostic variability, this review seeks to promote more consistent classification, better comparability across studies, and continued progress toward standardized assessment protocols. Convergence and vergence anomalies are addressed only when relevant as potential confounders or differential diagnostic considerations.

## Review

Amplitude of accommodation

Amplitude of accommodation represents the maximum dioptric change the eye can exert. It declines progressively with age and is a central parameter in diagnosing accommodative insufficiency and fatigue. Accommodative amplitude (AA) is the range over which the eye can change its optical power. This ability is present from birth, improves rapidly in infancy, and declines steeply after 20 years of age, with near-complete loss by 50-55 years [2,8,9]. AA is a key diagnostic tool for accommodative dysfunctions. Low AA may lead to greater accommodative effort during near work and is associated with reduced parasympathetic activity but strong sympathetic innervation [10,11]. AA can be measured via various subjective and objective methods, with results influenced by factors like depth of field, illumination, and pupil size. Diurnal variation in AA has also been observed [8].

Monocular Versus Binocular Amplitude of Accommodation

The neural control of accommodation, originating in the midbrain, suggests a fundamentally symmetric drive to both eyes, implying identical accommodative changes [9,12]. In practice, however, this symmetry can be disrupted. Factors such as a close working distance, a large interpupillary distance, a head tilt, or the presence of anisometropia can introduce significant intraocular accommodative asymmetries [12].

The viewing condition, monocular or binocular, also critically influences the response. A study by Vincent et al. on young adults revealed that while monocular viewing elicits symmetric accommodative behavior between the dominant and non-dominant eye, a shift occurs under binocular conditions [12]. In this more natural state, the dominant eye was observed to lead with a greater accommodative response. This finding is complemented by work from Park et al., which demonstrated that the accommodative response is both more accurate and efficient under binocular stimulation, achieving 90.9% of the actual demand compared to 84.6% monocularly [9]. Their analysis concluded that the binocular response is a composite of approximately 90% true accommodation and 10% pseuso-accommodation, a phenomenon where depth-of-field and other cues compensate for a slight refractive shortfall. A key principle emerging from this research, as noted by Koh and Charman, is that the binocular system tends to be guided by the eye that requires the lesser accommodative effort [13].

Dynamic accommodation

Dynamic accommodation describes the temporal behavior of the ocular accommodative response, i.e., changes in the eye’s refractive power (in diopters) over time, elicited by a time-varying accommodative demand (typically specified by target vergence and/or optical defocus) and commonly analyzed within a control-systems framework. It is typically quantified using step and sinusoidal paradigms: step changes in demand yield response timing and kinematics (e.g., latency, rise time, time constants, peak velocity), whereas sinusoidal modulation is characterized by frequency-response metrics such as gain (response amplitude relative to stimulus amplitude) and phase lag (temporal delay relative to the stimulus) [14-16]. Even under nominally steady viewing conditions, accommodation remains dynamic due to accommodative microfluctuations around the mean response, which are routinely analyzed as indices of response stability and the noise/bandwidth properties of the accommodative control system [17,18].

Dynamic accommodation is clinically and scientifically useful because it captures not only how much the eye can accommodate, but also how effectively the system operates in time, how rapidly it initiates and completes focus changes, how accurately it tracks changing demands, and how stable it remains during sustained near work [14,15,19]. These temporal properties are directly relevant to everyday visual tasks (reading, screen use, frequent near-far transitions), where sluggish dynamics and increased response variability can plausibly contribute to transient blur and asthenopic complaints; therefore, objective assessment of accommodative response and its dynamics can improve functional characterization of accommodative dysfunction beyond static amplitude measures alone [19]. Moreover, dynamic metrics are sensitive to short-term adaptation, including measurable changes in accommodation, accommodative vergence, and disparity vergence facility after repeated focus-shift cycles, making them suitable outcome measures for monitoring interventions and experimental manipulations [20]. Finally, measuring accommodative dynamics matters in display engineering because conflicts between vergence and accommodation can reduce visual performance and lead to eye strain or discomfort, especially when those conflicts change more quickly. [21,22].

Accuracy of accommodation, lag, and lead

The accuracy of the eye's focusing system is assessed by measuring the accommodative response to a given stimulus. It is a well-established physiologic norm that the response during near viewing is slightly less than the stimulus, a condition known as accommodative lag. A modest lag provides a degree of flexibility to the system and is associated with visual comfort. In contrast, when the accommodative response may exceed the level required for the near stimulus, a condition known as lead of accommodation [23-25].

Accommodative lag is formally defined as the shortfall in dioptric power when the eye's response is compared to the requirement for a specific stimulus distance [26,27]. It is most commonly measured clinically using dynamic retinoscopy techniques, such as the Nott retinoscopy, monocular estimated method (MEM), or with near cross-cylinder tests (fused/unfused cross-cylinder). Typical values are widely reported as 0 to +0.25 D for distance and +0.25 to +0.75 D for near targets (33-40 cm) [23,25-28]. Accommodative lag generally increases beyond this range with advancing age and with greater accommodative demand [25,26]. When lag consistently exceeds +0.75 D (at 33-40 cm), it often signals a clinical issue such as accommodative insufficiency, convergence excess, or an inaccurate refractive correction (e.g., over-minused myopia or under-corrected hyperopia) [25]. Less commonly, the accommodative response may exceed the level required for the near stimulus, a condition known as lead of accommodation, which may be related to disorders such as accommodative excess or convergence insufficiency [25].

The behavior of the accommodative reflex is dynamic and varies with stimulus demand; younger individuals frequently exhibit a slight accommodative lead (an over-response) at very low demands, which transitions to a lag as the demand increases [10]. Furthermore, the lag is not a fixed value but is influenced by task complexity and cognitive load. Mihelčič and Podlesek showed that short-term cognitive load affects accommodative behavior during near tasks; specifically, accommodative fluctuations were greater in tasks requiring logical reasoning [29]. Tosha et al. reported that individuals with higher visual discomfort showed an increase in accommodative lag over time during near viewing, whereas those with lower discomfort exhibited a more stable accommodative response [30].

Research into age-related trends of accommodative accuracy reveals a complex picture. Anderson et al. observed a decrease in lag with age in a cohort aged 3-20 years [31]. In contrast, Whitefoot and Charman, across 221 subjects aged 10-80 years, found a significant positive age-related trend in dynamic retinoscopy low neutral values, increasing at 0.034 D per year [32]. This discrepancy highlights methodological variations and the non-linear nature of accommodative development and decline. For instance, Alejandro et al., in a large sample, found no significant change in lag below 40 years of age, but a marked increase thereafter as the presbyopic transition began, with the accommodative response approaching zero around 54 years [26]. The influence of refractive error is also evident, as shown by Gwiazda et al., who reported higher mean lags in myopic children compared to their emmetropic peers [33].

A variety of clinical methods are available for measuring the accuracy of accommodation, with MEM retinoscopy and Nott dynamic retinoscopy being among the most frequently used techniques in clinical practice. The choice of measurement technique itself may impact the recorded values. García et al. compared MEM and Nott dynamic retinoscopy in 34 patients with vergence and accommodative disorders and found statistically significant differences between the two methods, with MEM values being more plus than Nott values [24]. Another study aimed at evaluating the agreement between accommodative lag measured using the MEM retinoscopy and Nott retinoscopy, in comparison with open-field autorefraction. The mean (standard deviation) lag for each technique was recorded as follows: MEM = 0.69 D, Nott = 0.62 D, autorefraction in the 180° meridian = 0.66 (0.50) D, and spherical-equivalent autorefraction = 0.60 D. The mean differences between techniques were small, ranging from −0.14 to +0.06 D, indicates that the average agreement between autorefraction and retinoscopic techniques is centered around zero (no systematic bias) in both children and adults [25].

Measuring accommodative ability and normal values

Accommodative Amplitude (AA)

AA is the maximum accommodative effort an individual can exert. It declines with age, making near tasks progressively more difficult. AA should be similar between eyes and is typically slightly lower monocularly than binocularly. Significant asymmetry warrants investigation into potential causes like medication, trauma, or neurological issues [34].

Hofstetter's formulas (Table 1) provide age-based regression estimates for AA [19], but recent studies consistently report AA values lower than these predictions across various populations, particularly in children [35]. This has led to proposals for revised, population-specific normative equations.

**Table 1:** Hofstetter's formulas D, diopters

Amplitude of accommodation measure	Hofstetter formula
Minimum amplitude of accommodation (D)	15 − (0.25 × age)
Average amplitude of accommodation (D)	18.5 − (0.30 × age)
Maximum amplitude of accommodation (D)	25 − (0.40 × age)

Subjective methods for AA

There are three subjective methods. (i) Push-Up Method: The most common clinical technique [19,34,36]. A target is moved toward the patient until sustained blur is reported. It is subjective and tends to overestimate AA due to depth-of-field effects. (ii) Push-Down Method: A target starts blurred and is moved away until clear. It typically yields smaller AA values than push-up. Averaging the results from both methods may provide a better estimate of true AA [18,34]. (iii) Minus-to-Blur: Negative lenses are added over a fixed near target until blur is reported. This method is fast and repeatable, but tends to underestimate AA [19].

Objective methods

Static accommodation reflects the accommodative response measured for a fixed target at a given moment, whereas dynamic accommodation describes how the visual system adjusts focus over time in response to changing visual demands. Dynamic assessment can provide clinically relevant parameters such as latency, response time, velocity, and accommodative microfluctuations, offering information beyond conventional static measures such as amplitude and lag of accommodation. Because visual tasks in everyday life involve continuous shifts in focus rather than fixed demands, dynamic objective assessment may provide a more functionally relevant characterization of accommodative behavior, particularly in patients with suspected accommodative dysfunctions [37]. Objective, device-based assessment is considered the most accurate method for evaluating dynamic accommodation, offering superior sensitivity, repeatability, and precision. Hammer et al. showed that dynamic stimulation aberrometry enables objective, high-time-resolution assessment of accommodation and revealed age-related reductions in objective accommodative amplitude together with longer response latency in older participants [38]. Separately, Kanclerz et al. reported that objective autorefractor-based measurements of accommodative amplitude were significantly lower than those obtained with conventional subjective methods [39]. A key consideration is that accommodative lag measurements vary with both the level of accommodative demand and the method or metric used to measure them, with greater lag typically found at higher accommodative demands when methods use a fixed small pupil diameter [40]. An objective dynamic assessment of accommodation may provide deeper information to characterise and understand the accommodative behaviour of subjects with accommodative dysfunctions; such objective measurements may also support clinical decision-making by helping clinicians differentiate between accommodative disorders, select appropriate management strategies, and monitor treatment outcomes over time.

Dynamic retinoscopy

An objective method assessing the accommodative response to a near target. It typically yields the lowest AA values but offers high inter-examiner repeatability and is not influenced by depth of field. It is invaluable for non-communicative patients or when subjective reliability is poor [32,34,41]. Dynamic retinoscopy can be performed using several techniques, including the Sheard technique, the Cross method, Nott dynamic retinoscopy, and the MEM. Among these, MEM retinoscopy has received the greatest attention and is generally considered to provide an accurate assessment of the accommodative response. It involves estimating the monocular accommodative response under binocular viewing conditions by briefly introducing spherical lenses in front of the spectacle plane. The Nott method is very similar to MEM retinoscopy. Instead of using lenses to achieve neutrality, the examiner moves the retinoscope closer or farther away until a neutral reflex is observed, while the patient fixates on a near target held at a constant distance. In contrast, with the Cross retinoscopy technique, the examiner adds spherical lenses binocularly to achieve neutrality while the patient focuses on a near target [19,23-25].

Consequently, it is of critical importance to compare objective and subjective (Table 2) measurements of accommodative amplitude and to establish updated normative reference values that account for age and ethnic variations.

**Table 2:** Clinical assessment of accommodative function: methods, outputs, limitations, and recommended use AA, amplitude of accommodation; MEM, monocular estimated method; FCC, fused cross cylinder; MAF, monocular accommodative facility; BAF, binocular accommodative facility; NRA, negative relative accommodation; PRA, positive relative accommodation; CPM, cycles per minute; D, diopters

Domain	Method	Output	Main systematic bias/Limitation	Best clinic use
Amplitude	Push-up/push-down	Subjective AA (D)	Push-up tends to overestimate AoA (depth-of-field); push-down typically yields lower values; endpoint variability [19,34,36]	Screening; quick AoA estimate
Amplitude	Minus-lens to blur (fixed distance)	Subjective AA (D)	Tends to underestimate AoA; depends on target/illumination and blur criterion [19,34]	More controlled AoA across visits
Response (static)	MEM/Nott/FCC	Lag/lead (D)	Method-dependent; examiner skill dependent; MEM introduces lenses (can bias neutrality) vs Nott (movement method) [24]	Differential diagnosis of accommodative response
Agreement	Autorefraction vs retinoscopy (open-field)	Lag/lead agreement	Recorded lag differs by technique; agreement varies across MEM/Nott/autorefraction depending on protocol [23-25]	When you need cross-method comparability/validation
Supportive	NRA/PRA	Relative ranges (D)	Endpoint criteria and broader binocular factors can influence results; supportive rather than standalone [24,42-45]	Complement AoA + facility in classification
Flexibility	MAF/BAF (±2.00 D)	cpm + failure side	Performance is criterion- and protocol-dependent; failure side (±) informs subtype [24,43,44]	Phenotyping: stimulate vs relax difficulty
Objective/ dynamic	Device-based dynamic accommodation (e.g., wavefront)	Latency/velocity/microfluctuations	Device/protocol dependent; results may deviate from classical norms; task demand affects lag/lead. [25,40,41]	Research-grade phenotyping; instability/fatigue profiling

Additional diagnostic tests for classifying accommodative dysfunctions

Accommodative disorders are heterogeneous, often requiring multiple tests for accurate diagnosis (Table 3). Positive relative accommodation (PRA), negative relative accommodation (NRA), and accommodative facility are key complementary assessments.

**Table 3:** Suggested clinical reference values synthesized from repeatedly reported norms across studies Normative values vary with age, refractive status, and testing method; values shown represent commonly cited clinical averages. Intended for orientation rather than strict cut-offs. MAF, monocular accommodative facility; BAF, binocular accommodative facility; CPM, cycles per minute; MEM, monocular estimated method; FCC, fused cross cylinder; D, diopters

Diagnostic Test	Expected/Normal Findings
MAF, ages 13–30 years	11 cpm [43,44,46,47]
MAF, ages 8–12 years	7 cpm [43,44,46,47]
BAF	Should not differ by more than 4 cpm from the MAF value
MEM or FCC – near lag of accommodation	+0.25 D to +0.75 D [23,24,32,46,47]
Negative Relative Accommodation	+2.00 D [42-44,47]
Positive Relative Accommodation	–2.25 D [42-44,47]
Amplitude of Accommodation	18 – (0.3 × Age in years) D

NRA

This test measures the ability to relax accommodation by adding plus lenses binocularly over a near target. Normative values are typically +1.75 to +2.50 D [28,42-44]. 

PRA

PRA assesses the ability to stimulate accommodation by adding minus lenses. Normative values are generally -2.00 to -2.50 D [43,44]. Low PRA (≤ -1.50 D) often suggests accommodative insufficiency, while very high PRA may indicate a condition of excess accommodation. A study by Yekta et al. in a young adult population with normal binocular vision reported a mean NRA of +2.08 D and a mean PRA of -2.92 D, noting variations by refractive error [42]. 

Accommodative Facility Testing

This test evaluates the ability to rapidly change focus using ±2.00 D flippers at near, measured in cycles per minute (cpm). Norms are approximately 7 cpm for ages 8-12 and 11 cpm for ages 13-30 monocularly (MAF). Binocular facility (BAF) should not differ from MAF by more than 4 cpm [43,44]. The test can also help detect vergence issues or suppression.

Accommodative dysfunctions

The accommodative mechanism may manifest through a range of functional abnormalities that compromise the ability to maintain clear and comfortable vision. Based on the Duke-Elder classification, the major accommodative dysfunctions comprise accommodative insufficiency, accommodative spasm, accommodative inertia, ill-sustained accommodation (accommodative fatigue), and accommodative infacility. Although these conditions differ with respect to their clinical characteristics, underlying pathophysiology, and diagnostic features, all of them may give rise to symptoms including asthenopia, intermittent blurred vision, diminished reading stamina, and difficulty with prolonged or alternating near visual tasks.

Accommodative Insufficiency

Accommodative insufficiency represents one of the most prevalent accommodative disorders, characterized by an inability to generate or sustain an adequate accommodative response for near vision in non-presbyopic individuals [19]. While typically non-pathological, it can co-occur with conditions affecting parasympathetic innervation. The diagnosis is primarily established when the AA is reduced by more than 2.00 D below the Hofstetter lower age-expected limit [19,36,47]. However, a comprehensive diagnosis should not rely on AA in isolation; it must be supported by a combination of other clinical signs. These include an abnormally high accommodative lag (>+0.75 D), failure on MAF testing with ±2.00 D lenses (less than 6 cpm), failure on BAF testing (less than 3 cpm), and a low PRA finding (< −1.25 D) [44,47]. The application of multiple criteria significantly refines diagnosis; one study showed prevalence dropped from 41.95% using one sign to just 2.93% when three signs were required [19].

Reported prevalence rates vary dramatically, from approximately 1% to 61.6%, largely reflecting heterogeneous populations and a critical lack of standardized diagnostic protocols across studies [19,46]. In children and adolescents, estimates range from 8% to 17% [19,46].

The symptomatology of AI is predominantly asthenopic. Patients commonly report blurred vision at near, headaches, eye strain, difficulty concentrating, poor academic or work performance, and avoidance of near tasks [19,46,47]. Non-ocular symptoms, including neck pain and poor posture, have also been associated.

Accommodative Infacility

Accommodative infacility describes a dysfunction in the ability to rapidly, accurately, and comfortably alter accommodative focus between different distances (e.g., far-to-near and back again) while maintaining appropriate vergence. This impairment in the flexibility of the accommodative system can significantly impact tasks requiring frequent shifts in viewing distance. Prevalence data are limited, but one study among Nepali students reported a rate of 2.85% [43].

Clinical assessment is typically performed using ±2.00 D flipper lenses. Normative values, as established by Scheiman and Wick, suggest that children aged 6-12 years should manage 5-6 cpm monocularly (minimum 3 cpm), while individuals aged 13-30 years should achieve around 11 cpm monocularly [47]. A key diagnostic indicator is a binocular facility rate that is more than 4 cpm slower than the monocular rate. This dysfunction is often suspected in children who exhibit notable difficulty or slowness when copying text from a whiteboard to a desk.

Accommodative Paresis/Paralysis

This condition involves a complete (paralysis) or partial (paresis) loss of accommodative ability due to a disruption in the neurological or muscular pathways. Etiologies are diverse and include systemic medications (e.g., anticholinergics), ocular or systemic pathology, trauma, intracranial tumors, or other neurological insults. It frequently presents as part of an oculomotor nerve (cranial nerve III) palsy, which is accompanied by other signs such as a non-reactive mydriasis, limitations in ocular motility, and a manifest deviation in primary gaze.

Accommodative Spasm (Pseudomyopia)

During accommodation, the ciliary muscles constrict in size, allowing a clear image to be focused on the fovea. The midbrain supranuclear impulse travels to the Edinger-Westphal nucleus to generate the motor command. The oculomotor nerve III transmits the motor command and is the synkinesis of three components. They are lens shape changes (accommodation), eye convergence, and pupil constriction (miosis). When any reflexes fail, the images become blurry and distorted. This condition is known as accommodative spasm [48,49]. The spasm can be categorized based on the components of the near triad involved. The most common form is isolated accommodative spasm. This can be followed by a spasm involving all three elements (accommodation, convergence, and miosis). A rare form is dominated by convergence abnormalities.

The etiology is often functional, representing a biological adaptation to sustained near-visual demands and psychological stress, mediated by parasympathetic overactivation via the Edinger-Westphal nucleus [46-48]. Precipitating factors most commonly include prolonged near work, which leads to sustained ciliary contraction with impaired relaxation, and psychological factors such as anxiety and stress [48-50].

From a refractive and binocular perspective, spasm can also arise in response to uncorrected hyperopia and significant exophoria. In an attempt to compensate for the exophoria and achieve single binocular vision, the accommodative system is driven to engage accommodative convergence, which in turn induces a pseudomyopic shift at distance. Common findings include: fluctuating distance and near visual acuity, blurred vision, diplopia, asthenopia, frontal headaches, micropsia or macropsia, and a significant discrepancy between subjective and objective refraction. Retinoscopy often reveals an inconsistent or "scissoring" reflex. Clinical testing typically shows an accommodative lead on MEM retinoscopy, very poor performance with ±2.00 D flippers (both MAF and BAF), and a low PRA (less than −1.50 D). The diagnosis is often confirmed by a significant reduction or elimination of the myopic shift after cycloplegic refraction [43,47-50].

Ill-Sustained Accommodation (Accommodative Fatigue)

Ill-sustained accommodation, also referred to as accommodative fatigue, is an accommodative dysfunction characterized by an initially adequate near-focus response that cannot be maintained over time. Individuals with this condition demonstrate normal accommodative ability at the onset of a near task but experience a progressive decline in clarity as the accommodative system fatigues [36,47]. Clinically, this breakdown in sustained accommodation manifests as intermittent near blur, reduced reading endurance, headaches, and the need for frequent refocusing or visual breaks. The disorder is often detected through accommodative facility testing or prolonged near-point evaluations, which reveal a marked deterioration in accommodative performance despite normal baseline measures. Ill-sustained accommodation is particularly relevant in school-aged populations, for whom sustained near work is essential, and it may contribute to reading difficulties, decreased comprehension, and overall visual discomfort during academic tasks [36,47].

Literature-derived diagnostic criteria for accommodative dysfunctions

The considerable variability identified across studies in diagnostic signs, testing protocols, and threshold values highlights the absence of universally accepted clinical criteria for accommodative dysfunctions. While individual authors and research groups have proposed useful diagnostic markers, these criteria often differ in emphasis and cut-off values, limiting comparability between studies and consistency in clinical practice.

To address this gap, the framework described in Table 4 was derived from a qualitative synthesis of the studies reviewed for this manuscript, with a focus on the diagnostic signs that have been repeatedly described throughout the most commonly referenced clinical texts. There was a preference for the data that was consistently reported throughout multiple studies and was readily available for use in a clinical setting, including accommodative amplitude, accommodative lag/lead, monocular and binocular accommodative facility, and relative accommodations. These values are intended for use as a general framework for clinical purposes, rather than being universally validated values, and the framework described is intended for use in a structured multiple sign classification system, not for replacing the judgment of the clinician.

**Table 4:** Diagnostic guidelines for accommodative dysfunctions CPM, cycles per minute; PRA, positive relative accommodation; D, diopters; AA, accommodative amplitude; NRA, negative relative accommodation; MAF, monocular accommodative facility; BAF, binocular accommodative facility

Disorder	AA [19,35,36,45,47,51]	Lag/lead [23-25,32,40,46,47]	MAF/BAF [19,46,47,51-53]	NRA/PRA [42,44-47,54]	Typical pattern
Accommodative insufficiency	low (>2 D below expected; Hofstetter)	High Lag (>0.75 D)	low (<6 cpm); often fails −2.00D	Low PRA (< −1.25 D)	Difficulty stimulating accommodation
Accommodative infacility	Often normal/mild lower	Normal or mild-higher lag	low; failure side varies by subtype	Variable	Difficulty switching focus (stimulate and/or relax)
Accommodative excess/spasm	Often normal; blur fluctuates	Lead and/or instability	low; often fails +2.00D	Variable	Difficulty relaxing accommodation
Ill-sustained / fatigue	Baseline near-normal	Drift/instability with sustained near	Drops with time-on-task	May reduce post-task	Deterioration during prolonged near work

Discussion and future directions

Current literature on accommodation shows significant methodological heterogeneity (Table 5).

**Table 5:** Reported diagnostic signs vary across studies AA, amplitude of accommodation; MEM, monocular estimated method; PRA, positive relative accommodation; NRA, negative relative accommodation; BAF, binocular accommodative facility; MAF, monocular accommodative facility; FCC, fused cross cylinder; D, diopters

Dysfunction	Study (Author, year)	Amplitude of Accommodation	MEM	Relative Accommodation	BAF	MAF
Accommodative insufficiency	Garcia et al., 2002 [45]	Reduced AA at least 2D below Hofstetter’s calculation for minimum amplitude: 15–0.25 X (age)	High MEM finding, > + 0.75 D	Low PRA, ≤ 1.25D	Fails binocular accommodative facility (BAF) with −2.00 D, ≤3 cpm	Fails monocular accommodative facility (MAF) with −2.00 D, ≤6 cpm.
	Scheiman and Wick, 2008 [47]	Low AA	High FCC OR MEM retinoscopy findings	Low PRA	Fails BAF with −2.00 D	Fails MAF with −2.00 D
	Hussaindeen et al., 2016 [53]	Reduced AA compared to the expected normal amplitudes for age as per the normative data	High MEM retinoscopy lag of accommodation (more than +1.25 DS).	-	Difficulty with BAF with − 2.00 DS	Difficulty with MAF with − 2.00 DS
Sustained Accommodation (Accommodative fatigue)	Darko-Takyi et al., 2016 [51]	Normal AA on first administration and decreases with repeated testing	High lag	Low PRA	Fails flipper test with minus lenses	Fails flipper test with minus lenses
Accommodative Infacility (Accommodative inertia)	Scheiman and Wick, 2008 [47]	Low AA	-	Low PRA and NRA	Fails BAF with accommodative flipper- 2.00 D	Fails MAF with accommodative flipper- 2.00 D
	Hussaindeen et al., 2016 [53]	-	-	-	Difficulty with BAF ±2.00 DS	Difficulty with MAF with ±2.00 DS Monocular accommodative facility < 7CPM
Accommodative Excess (Accommodative spasm)	Garcia et al., 2002 [45]	-	Low MEM retinoscopy finding, ≤0.25 D	Reduced NRA, ≤1.50 D, High PRA,≥-3.5 D	Difficulty clearing + 2.00 D with BAF, ≤3 cpm	Difficulty clearing + 2.00 D with MAF, ≤ 6 cpm
	Scheiman and Wick, 2008 [47]	-	Low MEM retinoscopy or FCC findings	Low NRA	Fails BAF facility with + 2.00 D	Fails MAF facility with +2.00 D
	Hussaindeen et al., 2016 [53]	-	Lead of accommodation	-	Difficulty clearing + 2.00 with BAF	Difficulty clearing + 2.00 DS with MAF with flipper

The accommodation literature is highly varied and inconsistent across different studies due to varying testing protocols and assessment methodologies, instead of true differences in the biology of the populations being studied. This heterogeneity has significant clinical implications, with reliance on individual clinically-based diagnostic measures such as AA or lag resulting in potential overestimation of prevalence and reduced diagnostic specificity. The use of multiple clinically based sign measures in a diagnostic classification system improves diagnostic accuracy and improves comparability between similar studies. Directions for future research to enhance the identification and understanding of accommodative dysfunctions should include:

Harmonisation of Protocols

Development and implementation of standardised examination procedures (e.g., target selection, work distance, criteria for determining blur, and criteria for determining accommodation facility) to reduce heterogeneous results and enhance consistency in evaluation between clinics and studies. 

Development of Normative Values

Future research should involve the establishment of age-, refractive error-, and ethnicity-specific standard values to provide more accurate diagnoses and to enhance our understanding of the differences between population-based variations in accommodative function.

Integration of Objective Dynamic Technologies

Incorporating advanced, device-based assessments-such as those measuring latency, velocity, and microfluctuations of accommodation-can provide deeper insights into the stability and responsiveness of the accommodative system. These technologies can serve as valuable tools for phenotyping, monitoring fatigue, and understanding the underlying physiology of accommodative anomalies.

Tighten Diagnostic Criteria

The inconsistent criteria currently available to diagnose patients indicate that strong, universally accepted criteria for testing are needed. Harmonized testing criteria will provide support for clinicians' diagnostic reliability and, therefore, support their clinical decision making. 

Reproducible Testing Methods and Integration of Patient Reported Symptoms

The use of reproducible testing methodologies combined with current computerised assessment techniques will give stronger support to the development of individualized and more effective treatment plans by helping to match patient-reported global symptoms with clinical signs. 

To summarize, the overall mission is to develop a standardized, evidence based assessment framework for accommodative dysfunction that utilises both established clinical signs with emerging objective technologies to help reduce inaccuracies in how patients are diagnosed/accommodative dysfunction; improve the amount and level of research comparability between studies that have similar clinical populations; and ultimately improve the overall outcome to patients who suffer from an abnormal functioning of the accommodation.

Limitations

This review has some limitations, mainly because of its narrative design and the wide variation among the available studies. Although major databases were searched, including PubMed, ScienceDirect, Wiley Online Library, and Scopus, it was not conducted as a formal systematic review, and no structured risk-of-bias assessment was performed. In addition, publication bias cannot be excluded. Furthermore, substantial heterogeneity existed across studies in terms of patient populations, refractive characteristics, testing methods, outcome definitions, and diagnostic thresholds, making direct comparison difficult and precluding any meaningful quantitative synthesis. Therefore, the diagnostic framework presented here should be interpreted as a practical, hypothesis-generating clinical guide rather than a universally validated, guideline-level classification system.

## Conclusions

Non-presbyopic patients with accommodative dysfunctions are often difficult to assess and classify due to considerable variability in testing procedures, criteria used in diagnosis, and norms used in testing. The variability is reflected in testing outcomes and may result in misclassification and comparability issues. The multi-sign diagnostic technique, which is based on the combined evaluation of essential signs used in diagnosis, may help in increasing the specificity of diagnosis and providing a more reliable basis for decision-making in patients with suspected cases of accommodative dysfunctions. Future research directions in this area are expected to result from the development of standardized testing procedures, the provision of reference values based on age, refractive errors, and population stratification, and the integration of objective dynamic technology into testing procedures.
